# Supplementary material for: Trend of Admissions Due to Chronic Lower Respiratory Diseases: An Ecological Study
Source: Healthcare (Basel). 2022 Dec 26;11(1):65. doi: 10.3390/healthcare11010065 (PMC9818740; doi:10.3390/healthcare11010065)
Supplement: Supplementary file 1 [file healthcare-11-00065-s001.zip › healthcare-2070390-supplementary.pdf]

Supplementary Material S1: Hospital admission rates for chronic lower respiratory diseases in England and Wales stratified by gender.

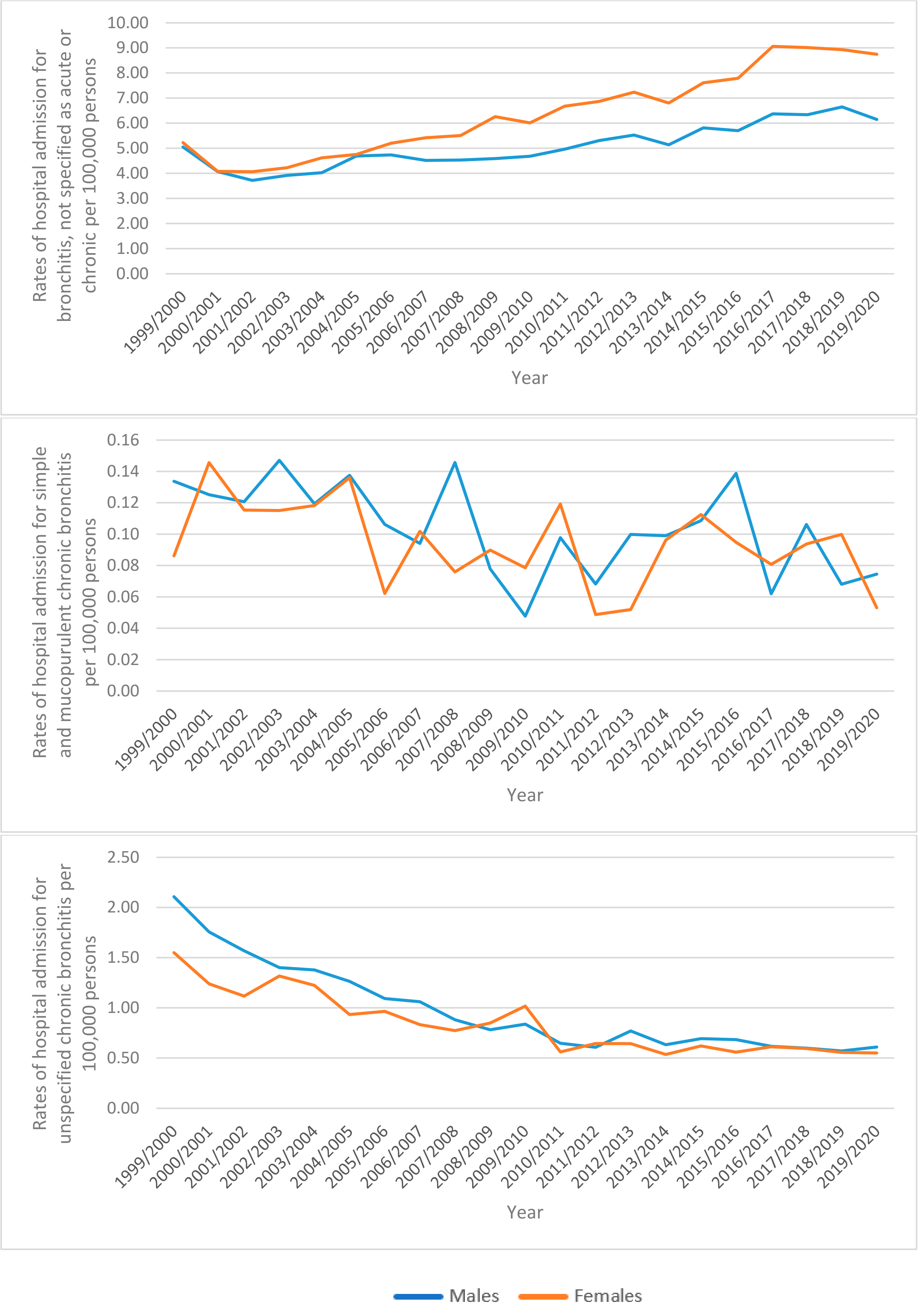

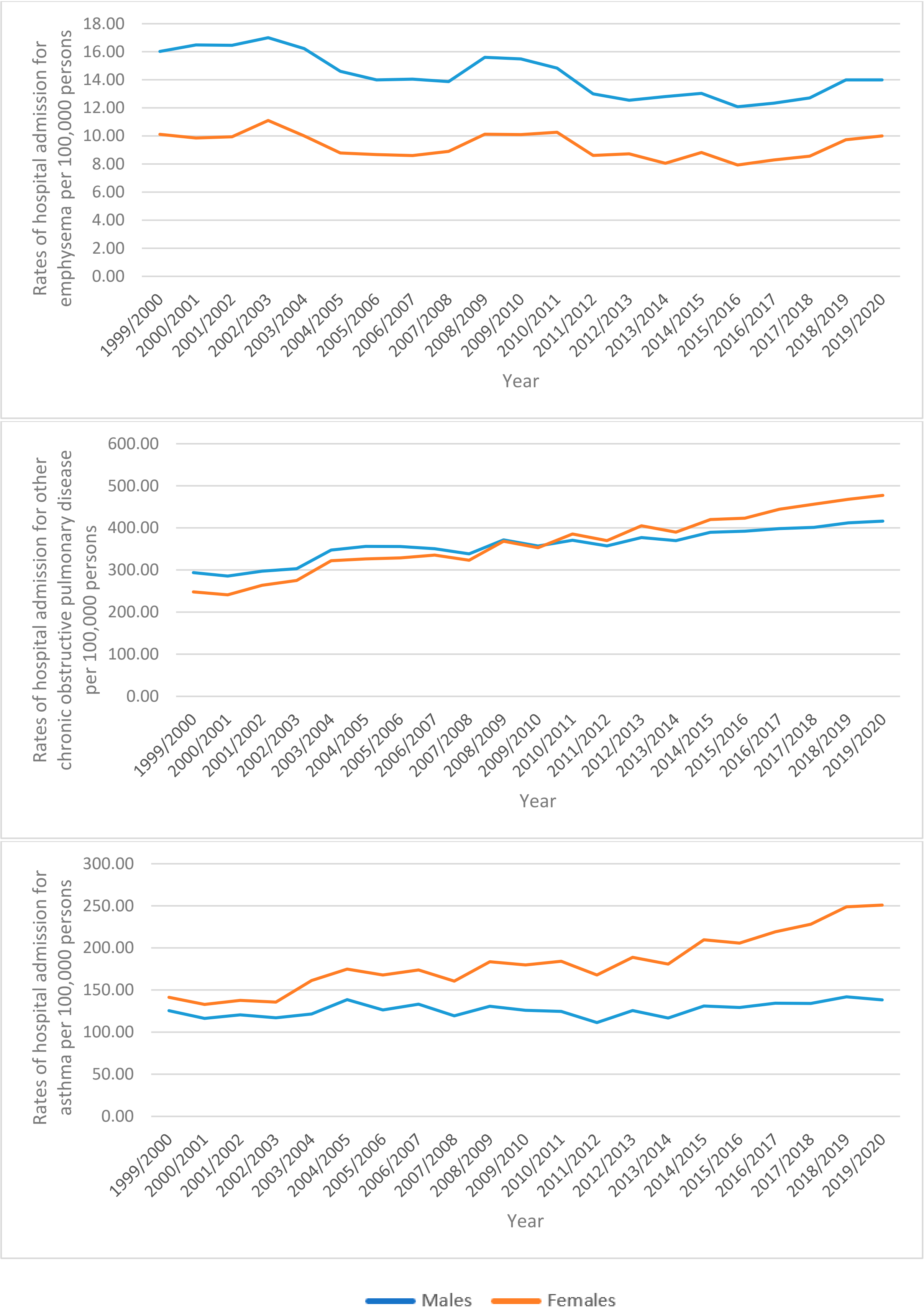
Rates of hospital admission for emphysema per 100,000 persons
18.00
16.00
14.00
12.00
10.00
8.00
6.00
4.00
2.00
0.00
1999/2000
2000/2001
2001/2002
2002/2003
2003/2004
2004/2005
2005/2006
2006/2007
2007/2008
2008/2009
2009/2010
2010/2011
2011/2012
2012/2013
2013/2014
2014/2015
2015/2016
2016/2017
2017/2018
2018/2019
2019/2020
Year
Rates of hospital admission for other chronic obstructive pulmonary disease per 100,000 persons
600.00
500.00
400.00
300.00
200.00
100.00
0.00
1999/2000
2000/2001
2001/2002
2002/2003
2003/2004
2004/2005
2005/2006
2006/2007
2007/2008
2008/2009
2009/2010
2010/2011
2011/2012
2012/2013
2013/2014
2014/2015
2015/2016
2016/2017
2017/2018
2018/2019
2019/2020
Year
Rates of hospital admission for asthma per 100,000 persons
300.00
250.00
200.00
150.00
100.00
50.00
0.00
1999/2000
2000/2001
2001/2002
2002/2003
2003/2004
2004/2005
2005/2006
2006/2007
2007/2008
2008/2009
2009/2010
2010/2011
2011/2012
2012/2013
2013/2014
2014/2015
2015/2016
2016/2017
2017/2018
2018/2019
2019/2020
Year
Males
Females

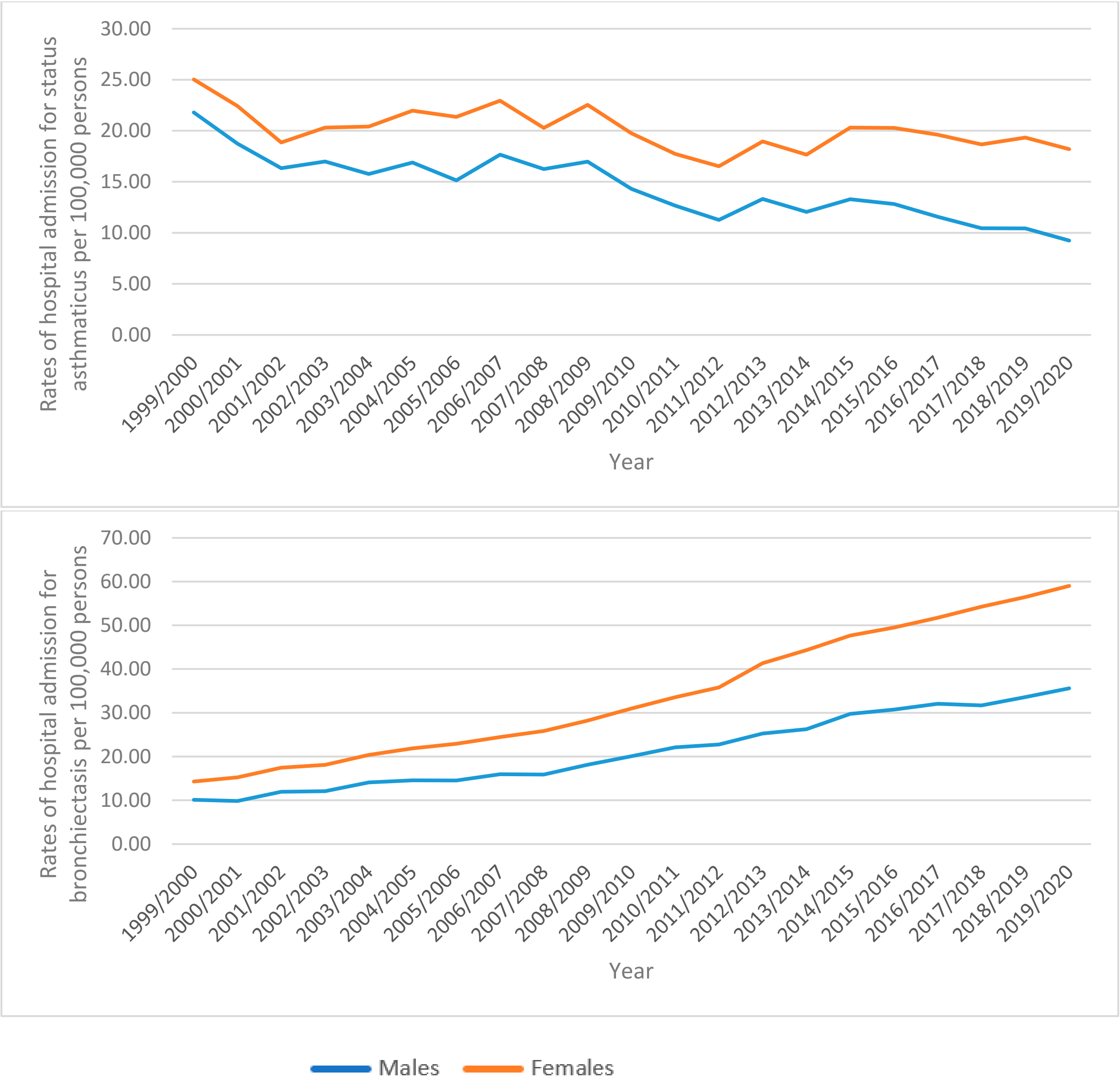

Rates of hospital admission for status asthmaticus per 100,000 persons
30.00
25.00
20.00
15.00
10.00
5.00
0.00
1999/2000
2000/2001
2001/2002
2002/2003
2003/2004
2004/2005
2005/2006
2006/2007
2007/2008
2008/2009
2009/2010
2010/2011
2011/2012
2012/2013
2013/2014
2014/2015
2015/2016
2016/2017
2017/2018
2018/2019
2019/2020
Year
Rates of hospital admission for bronchiectasis per 100,000 persons
70.00
60.00
50.00
40.00
30.00
20.00
10.00
0.00
1999/2000
2000/2001
2001/2002
2002/2003
2003/2004
2004/2005
2005/2006
2006/2007
2007/2008
2008/2009
2009/2010
2010/2011
2011/2012
2012/2013
2013/2014
2014/2015
2015/2016
2016/2017
2017/2018
2018/2019
2019/2020
Year
Males
Females

Supplementary Material S2: Hospital admission rates for chronic lower respiratory diseases in England and Wales stratified by age.

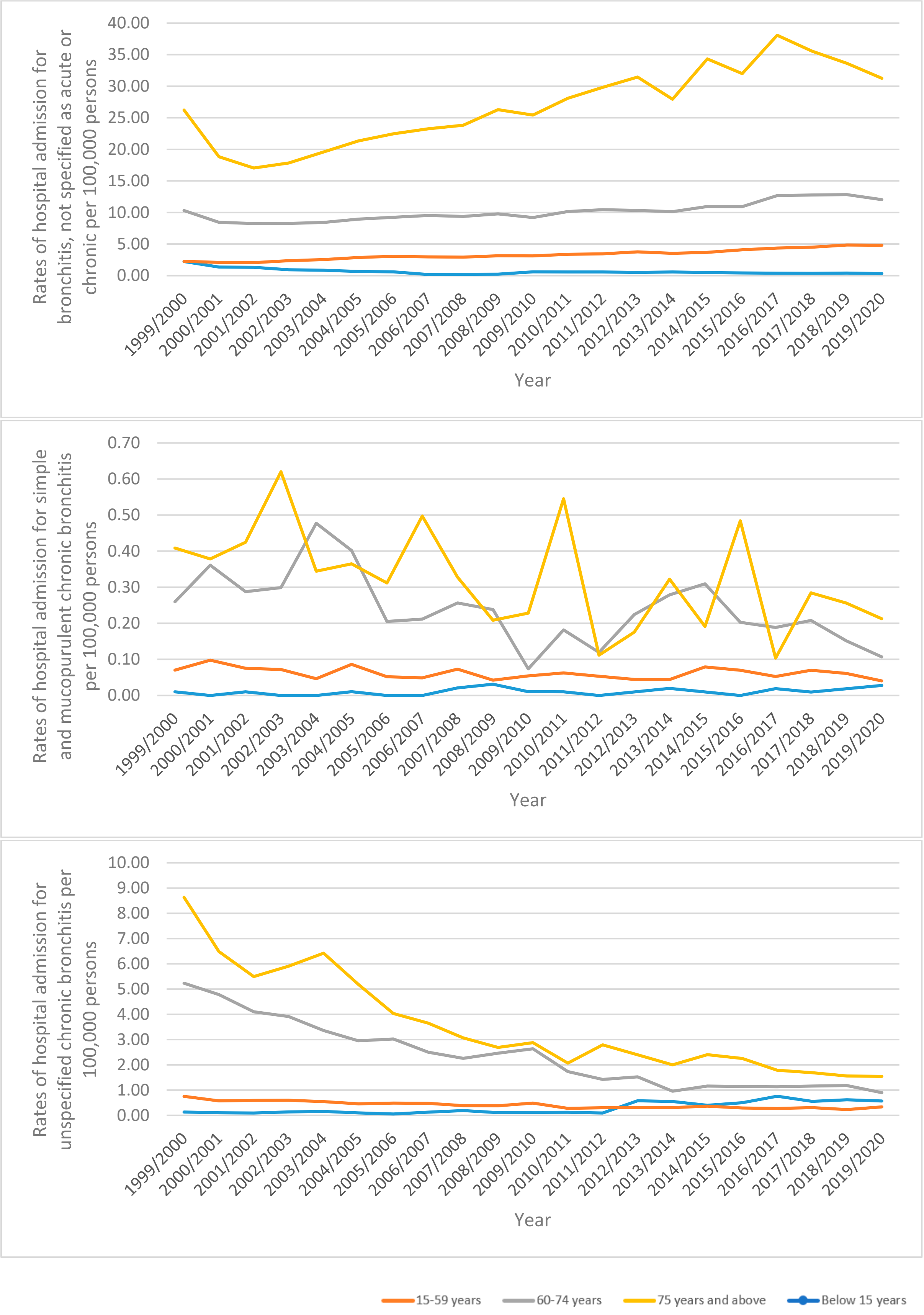

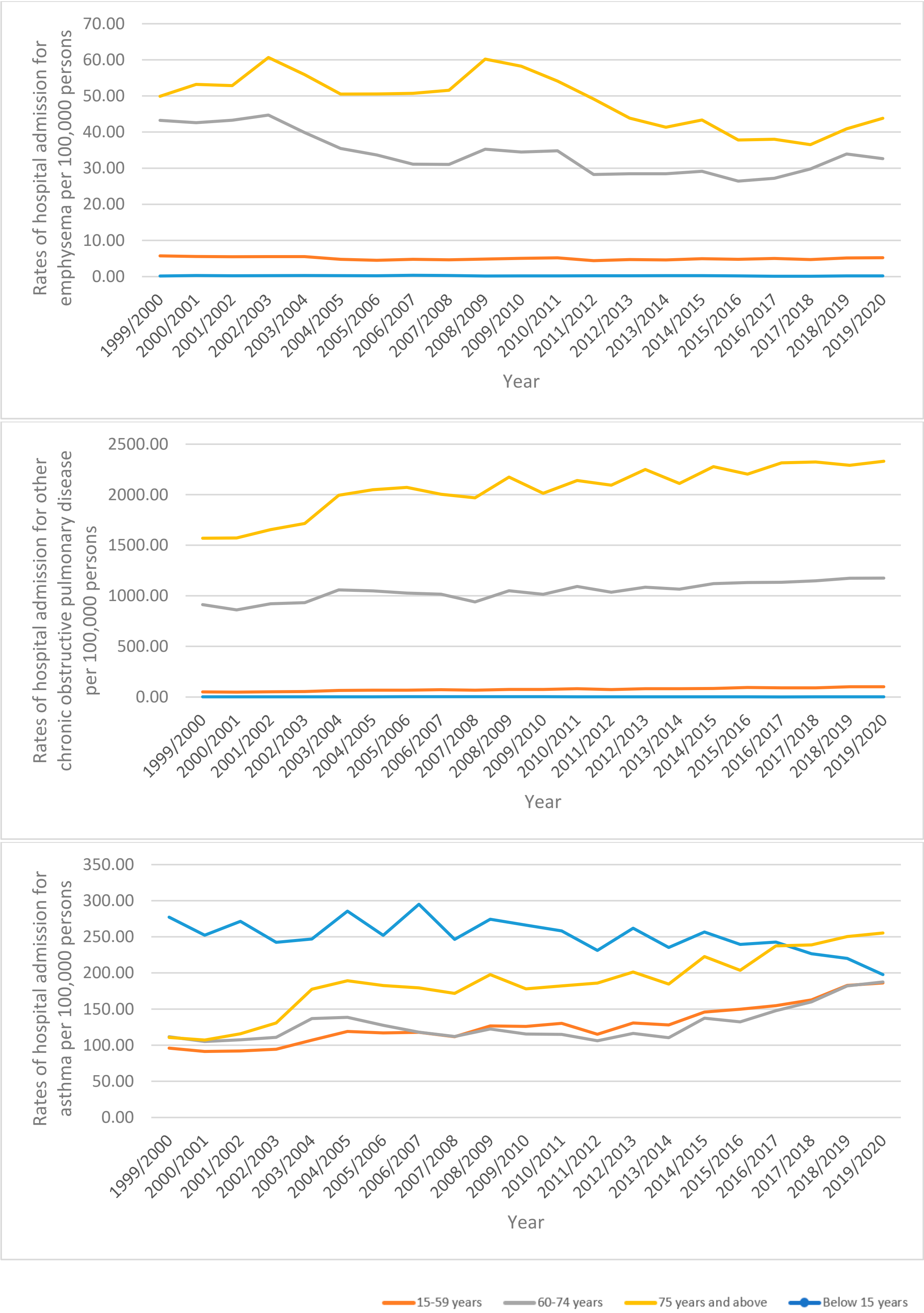
Rates of hospital admission for emphysema per 100,000 persons
70.00
60.00
50.00
40.00
30.00
20.00
10.00
0.00
1999/2000 2000/2001 2001/2002 2002/2003 2003/2004 2004/2005 2005/2006 2006/2007 2007/2008 2008/2009 2009/2010 2010/2011 2011/2012 2012/2013 2013/2014 2014/2015 2015/2016 2016/2017 2017/2018 2018/2019 2019/2020
Year
Rates of hospital admission for other chronic obstructive pulmonary disease per 100,000 persons
2500.00
2000.00
1500.00
1000.00
500.00
0.00
1999/2000 2000/2001 2001/2002 2002/2003 2003/2004 2004/2005 2005/2006 2006/2007 2007/2008 2008/2009 2009/2010 2010/2011 2011/2012 2012/2013 2013/2014 2014/2015 2015/2016 2016/2017 2017/2018 2018/2019 2019/2020
Year
Rates of hospital admission for asthma per 100,000 persons
350.00
300.00
250.00
200.00
150.00
100.00
50.00
0.00
1999/2000 2000/2001 2001/2002 2002/2003 2003/2004 2004/2005 2005/2006 2006/2007 2007/2008 2008/2009 2009/2010 2010/2011 2011/2012 2012/2013 2013/2014 2014/2015 2015/2016 2016/2017 2017/2018 2018/2019 2019/2020
Year
15-59 years
60-74 years
75 years and above
Below 15 years

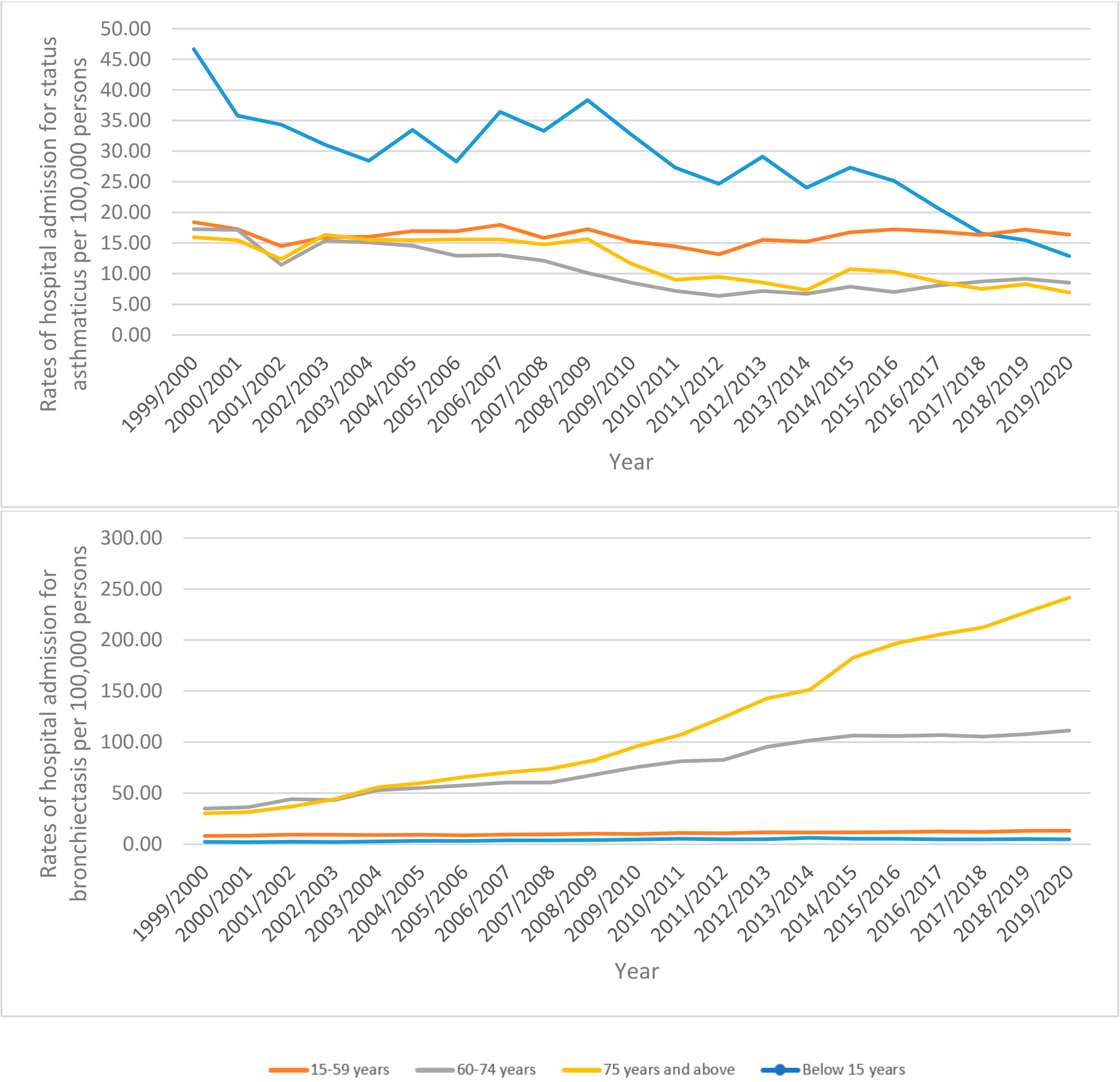
Rates of hospital admission for status asthmaticus per 100,000 persons
50.00
45.00
40.00
35.00
30.00
25.00
20.00
15.00
10.00
5.00
0.00
1999/2000
2000/2001
2001/2002
2002/2003
2003/2004
2004/2005
2005/2006
2006/2007
2007/2008
2008/2009
2009/2010
2010/2011
2011/2012
2012/2013
2013/2014
2014/2015
2015/2016
2016/2017
2017/2018
2018/2019
2019/2020
Year
Rates of hospital admission for bronchiectasis per 100,000 persons
300.00
250.00
200.00
150.00
100.00
50.00
0.00
1999/2000
2000/2001
2001/2002
2002/2003
2003/2004
2004/2005
2005/2006
2006/2007
2007/2008
2008/2009
2009/2010
2010/2011
2011/2012
2012/2013
2013/2014
2014/2015
2015/2016
2016/2017
2017/2018
2018/2019
2019/2020
Year
15-59 years
60-74 years
75 years and above
Below 15 years
